# Effects of lifestyle modification counselling on weight and body composition among adults with overweight or obesity on antiretroviral therapy: a randomized controlled trial

**DOI:** 10.1016/j.obpill.2025.100184

**Published:** 2025-06-06

**Authors:** Agete Tadewos Hirigo, Moges Derbe, Daniel Yilma, Ayalew Astatkie, Zelalem Debebe

**Affiliations:** a School of Medical Laboratory Science, College of Medicine Health Sciences, Hawassa University, Hawassa, Ethiopia; b Center for Food Science and Nutrition, Addis Ababa University, Addis Ababa, Ethiopia; c Department of United States Veteran Affairs, 10 N Greene St, Baltimore, MD, 21201, USA; d Department of Internal Medicine, College of Public Health and Medical Sciences, Jimma University, Jimma, Ethiopia; e Clinical Trial Unit, Jimma University, Jimma, Ethiopia; f School of Public Health, College of Medicine and Health Sciences, Hawassa University, Hawassa, Ethiopia

**Keywords:** Overweight/obesity, HIV, Antiretroviral therapy, Lifestyle, Counselling

## Abstract

**Background:**

we aimed to evaluate the impact of lifestyle modification counselling on weight and body composition among adults with overweight or obesity receiving antiretroviral treatment (ART).

**Methods:**

This randomized, open-label, controlled trial enrolled 126 adults aged 18–65 years, living with human immunodeficiency virus (HIV) and classified as overweight or obese. Of these, 116 participants (63 in the control group and 53 in the intervention group) completed the six-month follow-up. Weight and body composition outcomes were analyzed among those who completed the study, with comparisons made between baseline and post-intervention measurements. The effect of lifestyle modification counselling on weight and body composition was evaluated through linear mixed effects model and multiple linear regression analysis.

**Results:**

Over the six-month trial, participants who received lifestyle modification counselling showed significantly greater reductions in body weight (Δ = −1.4 kg, p = 0.002), visceral fat (Δ = −0.53, p = 0.006), fat mass (Δ = −2.16 kg, p ​< ​0.001), and body fat percentage (Δ ​= ​−2.02 ​%, p ​< ​0.001) compared to the control group. The intervention group experienced greater increases in fat-free mass (Δ ​= ​+0.673 kg, p ​= ​0.068) and percentage skeletal muscle (Δ ​= ​+1.27 ​%, p ​< ​0.001) compared to the control group. Additionally, lifestyle modification counselling significantly contributed to achieving at least a 3 ​% weight loss from baseline (β ​= ​0.158, p ​= ​0.048).

**Conclusion:**

Lifestyle modification counselling resulted in promising and favorable changes in weight and body composition. Therefore, offering routine structured counselling interventions within ART clinics for individuals with overweight or obesity may help reduce obesity-related health risks and improve clinical outcomes (Thai Clinical Trials Registry TCTR20240905007).

## Highlights

• Lifestyle counselling resulted in significant reductions in weight, fat mass, visceral fat and body fat percentage.
• The intervention group showed an increase in fat-free mass and skeletal muscle, reflecting improved body composition.
• Overall, 21.6 % of participants had at least a 3 % weight loss from their baseline.
• Counselling significantly correlated with ≥3 % weight loss.
• The findings indicate that lifestyle counselling leads to favorable changes in weight and body composition.

## Graphical abstract

**Figure:**
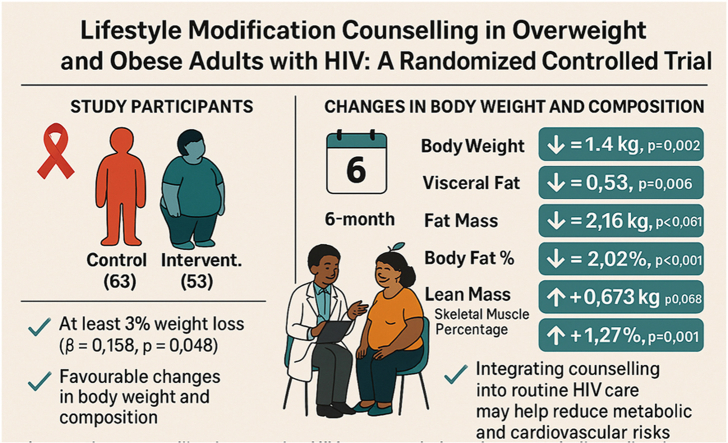

## 1 Introduction

Individuals living with human immunodeficiency virus (HIV) who are receiving antiretroviral treatment (ART) are more likely to experience obesity compared to the general population [1]. The weight gain observed in this group might be influenced by a variety of factors, which include the obesogenic situation, metabolic as well as immune changes associated with HIV, specific ART regimens and demographic variables [2]. Excess weight gain in people living with HIV (PLWH) is associated with an elevated risk of noncommunicable diseases (NCDs), which occur more frequently in this population compared to those without HIV [3,4]. Additionally, obesity in PLWH is considered as a risk factor for cardiovascular and metabolic disorders and certain cancers [5,6].

Recent studies have highlighted differential degrees of weight gain associated with various ART medications [7,8]. Integrase strand transfer inhibitors (INSTIs), particularly dolutegravir (DTG)-based regimens, are highly recommended by the World Health Organization (WHO) and widely used in Sub-Saharan Africa (SSA) due to their effectiveness, low resistance and safety [9]. Despite their established efficacy, DTG-based regimens have been associated with undesirable effects, including potential cardiometabolic risks [7,10]. In addition, emerging evidence suggests an increased risk of weight gain with these regimens [11,12] and prior findings from this study project showed that 50 % of participants initiating first-line ART gained ≥10 % of their baseline weight within 24-months [13]. A significant proportion of those who gained excess weight were on DTG-based therapies [13]. As a result, various studies have recommended the implementation of weight-loss interventions for PLWH on INSTIs-based regimens [[14], [15], [16]].

Studies have emphasized the importance of behavioral interventions, particularly dietary changes and regular physical activity as primary and non-pharmacological options for weight loss and managinig obesity-related conditions [17,18]. Robust evidence suggests that interventions combining caloric restriction with exercise can effectively facilitate healthy weight loss, leading to decreases in both subcutaneous and visceral fat among PLWH [[19], [20], [21]]. Importantly even modest reductions in weight can yield substantial long-term health advantages for adults with obesity in the general population [22,23], including cardiometabolic risk profiles [17]. Consequently, implementing lifestyle changes focused on weight reduction is crucial for individuals with overweight and or obesity, considering the substantial health risks that obesity poses to this group [24,25]. Regular lifestyle counselling represents an essential and cost-effective method to support healthy weight loss, sustainable management management and cardiovascular risk risk reduction in this population [71].

Although Ethiopia has began integrating NCD care into HIV services but lifestyle modification counselling is not consistently provided to PLWH who are overweight or obese and receiving ART. The increasing prevalence of weight gain associated with newer ART treatments, exacerbated by sedentary lifestyles and limited engagement in health-promoting activities, underscores the urgent need for wellcontrolled studies. This study aimed to assess the impact of lifestyle modification counselling on weight and body composition among adult PLWH with a body mass index (BMI) ≥25 kg/m^2^ who were receiving first-line ART. Using a randomized trial design, the study aimed to highlight the effectiveness of these interventions and their relevance to HIV care.

## 2 Materials and methods

### 2.1 Study setting

This study was done in Hawassa city in Sidama Region, southern-Ethiopia, located 275 km from Addis Ababa. Hawassa city covers an area of 157.2 km^2^, is divided into 8 sub-cities and consists of 32 kebeles (the smallest administrative unit or neighbourhood within a district or sub-city). According to the 2022 report from the City Administration Health Department, the city had an estimated population of 402,903, comprising 201,532 males and 201,371 females. The city administration was providing ART services to over 6300 PLWH through eight health facilities during the study period.

### 2.2 Study design and participants

This randomized, open-label, controlled trial (Thai Clinical Trials Registry TCTR20240905007) investigated the effects of behavioral lifestyle interventions in adults with overweight or obesity receiving ART. Randomization was performed using a simple lottery method based on the participants' arrival for routine ART follow-up visits. Participants were sequentially assigned to either the intervention or the control group, ensuring comparable age distribution across groups. The intervention group received personalized face-to-face counselling on behavioral lifestyle changes at baseline, followed by at least two phone-counselling sessions within two months to encourage adherence. In contrast, the control group received routine care from the ART clinics. Both study groups underwent baseline data collection prior to the intervention and were followed up for six months after the initial face-to-face counselling session.

To be eligible, PLWH had to have a BMI ≥25 kg/m^2^, be aged 18–65 years, and have been on first-line ART for at least 12 months following Ethiopia's test-and-treat strategy. Additionally, eligible participants had a baseline physical activity level below 600 metabolic equivalents of task per week (MET-min/week) and were free from opportunistic infections during the study period. Importantly, none had previously received formal dietary or physical activity counselling or participated in structured nutrition or physical activity programs.

Additionally, participants were required to have achieved viral suppression, defined as an HIV ribonucleic acid (RNA) viral load below 1000 copies/mL, and demonstrated at least fair adherence to ART (missing no more than 3–4 doses out of 30 or 4–9 doses out of 60) [26]. All participants provided written informed consent prior to enrolment. Their first-line ART regimen included two nucleoside/nucleotide reverse transcriptase inhibitors plus either efavirenz- or dolutegravir-based combinations.

Exclusion criteria included a history of pulmonary or cardiac complications, neurological or musculoskeletal disorders, diagnosed thyroid disease, uncontrolled hypertension, or any physical condition that limited participation in physical activity. Pregnant women, those with chronic liver or renal disease, and participants who had begun Ethiopian Orthodox Christian Lent and Ramadan fasting practices during the study period were also excluded. Study enrolment took place between January 5, 2023, and May 30, 2024.

### 2.3 Sample size determination

For this trial study, the OpenEpi 3.01 software was used to estimate sample size, with the focus on changes in body weight following lifestyle modification counselling. The calculation was based on a 95 % confidence interval (CI) and 80 % power. At the three-month assessment, the counselled group had a mean 85.6 kg (±15.96) weight, with mean 4.09 kg reduction from baseline [27]. While, the participants in control arm had an average weight of 94.02 kg (±14.96), indicating a mean 0.65 kg increase from baseline [27]. Initially, the sample size was calculated to be 106, but it was adjusted to 132 to account for a 20 % attrition rate. As a result, 66 participants were randomly assigned to each group, with randomization based on their arrival according to the ART clinic's follow-up schedule.

### 2.4 Intervention

The goal of the lifestyle modification intervention was to achieve and maintain at least a 3 % reduction in weight from baseline through individualized counselling, including an initial face-to-face session followed by a minimum of two phone-based remote counselling sessions. The intervention included specific strategies such as modifying dietary intake habit, caloric restriction and increasing body exercise (performing at least moderate-intensity physical activity). Trained counsellor (ART clinic nurse) conducted counselling. The training materials for counsellor were developed by adapting guidelines and literature on body weight management, emphasizing the importance of healthy weight reduction [[28], [29], [30], [31], [32], [33]]. The initial session was an individual based face-to-face contact, lasting 50–60 min, with a focus on promoting lifestyle changes. All intervention components were explained in Amharic language during counselling supported by pictures and photographs during face-to-face interviews and discussions. Each participant received nutrition counselling from a trained counsellor, who utilized motivational interviewing techniques overseen by the principal investigator. During the counselling sessions, counsellor encouraged participants’ lifestyle modifications to reduce caloric intake of at least 500 kcal/day. To achieve this they were advised to use strategies such as reducing portion sizes, limiting high-calorie foods (e.g., fried foods, sugary drinks), choosing low-energy-dense foods like vegetables and whole grains, and promoting regular meal patterns, lean protein and low fat and oils into their daily diets. In addition, they recommended incorporating locally available fibre-rich foods, like whole grains, green vegetables, Moringa *oleifera* leaves, and fruits into their diets. They also advised avoiding overeating and to reduce or eliminate the intake of refined grains, fats particularly saturated types, added sugars, high-calorie foods, fast foods and sugary or processed foods.

Regarding exercise, all participants in the intervention group were encouraged to engage in at least 150 min of moderate-intensity exercise per week, primarily consisting of aerobic activities. In addition to this, they were advised to perform physical exercises in line with the program aired on Ethiopian television, either choosing morning or evening sessions based on their preference. They were also highly advised to minimize sedentary behaviours, including prolonged sitting, watching television, using a computer, or playing video games [34,35]. Each participant in the intervention group received a brochure, written in Amharic language, which was adapted from multiple guidelines and previously published literature on body weight management [[28], [29], [30], [31], [32], [33]]. This brochure included both narrative text and pictorial illustrations offering guidance on dietary modifications, behavioural changes, and the promotion of physical activity to support effective weight loss. 10.13039/100014337Furthermore, participants received at least two remote counselling sessions via phone calls, each lasting 20–30 min within the first two months following randomization, to support and encourage them to adhere to the recommended lifestyle changes. In contrast, the control group have not received lifestyle modification counselling throughout the study but they were asked to visit and attend the final assessment. Apart from the counselling, participants received the routine care from the ART clinics service. Following the final assessment, they were provided with lifestyle modification counselling to enable them to achieve the anticipated benefits.

### 2.5 Data collection

Data on sociodemographic and socioeconomic factors, behavioral characteristics, clinical information, food frequency and 24-h ​dietary recall (24HR) were collected through a questionnaire written in the Amharic language administered by an interviewer. All data were gathered in isolated rooms according to the measurement protocol. To capture detailed information on all foods and beverages consumed by participants, a structured 24HR questionnaire was used. This covered everything consumed within 24 h, typically from morning of the previous day to the morning of the next day.

To estimate food intake, standard measuring tools, common-size containers and pictorial displays of food items were used. Photographs illustrating different portion sizes was used to facilitate precise estimation of the foods consumed and their respective portion sizes according to the participants' responses. This process enabled the calculation of average daily use of total calorie, macronutrients (carbohydrates, proteins and fats) and fibre amount. These estimates were computed using Ethiopian Food Composition Tables and Ethiopian Food-Based Dietary Guidelines [[36], [37], [38]]. A questionnaire on dietary diversity and food frequency was used to evaluate the participants' usual food intake. The number of distinct food groups consumed by participants over the past 24 h was used to determine the Individual Dietary Diversity Score (IDDS). For this assessment, food items were categorized into ten specific groups. A DDS of less than 5 indicated low dietary diversity, while a score of 5 or more reflected diversified food consumption [39,40]. In addition, the baseline physical exercise status of each individual was evaluated using the short form of the International Physical Activity Questionnaire [41,42].

### 2.6 Outcome measurement and assessments

The outcome variables were measured at baseline, before the initiation of counselling, and again at the end of the six-month intervention. Measurements of body height done by a Seca stadiometer from Germany, while waist circumference and hip circumference were measured with non-elastic measuring tape and recorded in centimetres (cm). Body weight in kilogram, BMI (kg/m^2^) and body composition including body fat percentage (BFP), visceral fat (rated on a scale of 1–30) and percentage of skeletal muscle were obtained through bioelectrical impedance analysis using the Omron Full Body Sensor Body Composition Monitor and Scale (Omron HBF–514C: Omron Healthcare Ltd.) [43], and measured as per manufacturers protocol. Fat mass (FM) was calculated by multiplying body weight by the BFP divided by 100, while fat-free mass (FFM) was determined by subtracting FM from total body weight, using the formula: FFM = weight − [weight × (BFP/100)]. The Fat Mass Index (FMI) is calculated by dividing FM by the square of height (m^2^), expressed as: FMI = FM (kg)/height (m^2^). Similarly, the Fat-Free Mass Index (FFMI) is determined by dividing FFM by the square of height (m^2^), represented as: **FFMI = FFM (kg)/height (m^2^).**

To ensure data accuracy, each outcome variable was measured twice for every participant at each phase and the mean of these values was recorded and used for analysis. The same counsellor provided the intervention consistently throughout the study to reduce interpersonal bias and body weight measuring instrument was calibrated daily with standard calibration tools.

The primary outcomes were changes in weight and body composition, measured at baseline and after six months. The the study also aimed to achieve at least a 3 % reduction in weight from baseline after six months of lifestyle modification counselling. This weight loss was expressed as a percentage change from baseline for each participant, using the formula: [(value at 6 months − value at baseline)/value at baseline] × 100.

### 2.7 Statistical analysis

To minimize biases from non-completers, we conducted a per-protocol analysis, comparing study groups based only on participants who completed the study as planned. Descriptive statistics were employed to summarize the study variables. Categorical variables are presented as frequencies and percentages, while numerical variables are reported as means with standard deviations (SD) or as medians with interquartile ranges (IQRs). The χ^2^ test was applied to evaluate the demographic characteristics of the participants and Independent *t*-test was used to compare baseline characteristics between the study groups. A paired *t*-test was used to calculate within-group changes in quantitative variables from baseline. An independent *t*-test was used to evaluate the differences in scores between the pre-test and post-test results for both the intervention and control groups. Demographic variables and weight loss were dummy coded before analysis. Multicollinearity was evaluated using the variance inflation factor (VIF), with values < 10 considered acceptable for this analysis. Linear regression, adjusted for baseline values was employed to assess changes in weight and body composition, as well as to determine a weight loss of at least 3 %. A linear mixed-effects model was also employed to assess the effect of counselling on weight and body composition variables over time. Statistical significance was determined at a 5 % alpha level with a 95 % CI. Analyses were performed using SPSS version 27.0 and STATA version 17.0.

## 3 Results

### 3.1 Participants’ baseline characteristics

**Fig. 1:**
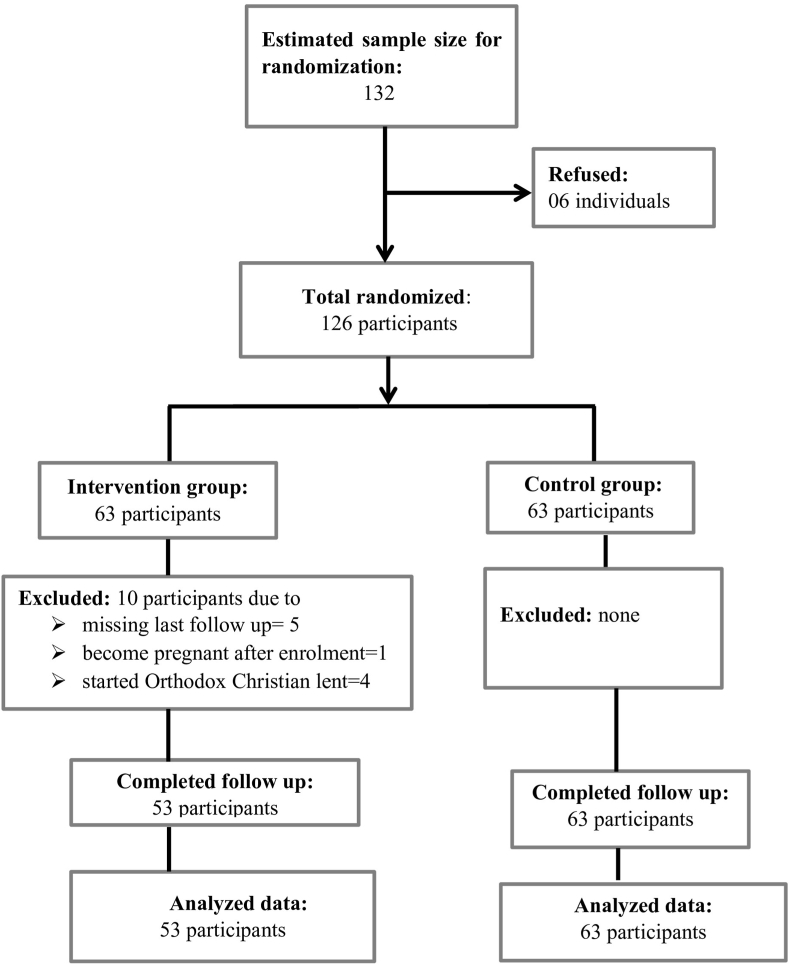
Flowchart depicting the study participants' inclusion process.

Overall, 126 adults with overweight and obesity were randomly allocated into two groups, each consisting of 63 individuals. Following randomization, 10 participants were excluded from the intervention group due to failure to complete the study (Fig. 1). Table 1 indicates the study completed participants’ baseline characteristics (n = 116). There were no statistically significant differences between these groups in baseline variables (all p > 0.05), except for dietary fibre consumption level. For both study groups, the average age was 43.6 years (SD ± 8.6), and the average BMI was 29.7 kg/m^2^ (SD ± 4.8). The majority were females (71/116, 61.2 %) and overweight (79/116, 68.1 %). Regarding ART regimens, more than 95 % in each group were on DTG based first line regimens.

**Table 1:** Baseline general characteristics of the study participants.

Variables	Category	Total n = 116	Study groups		p value
			Intervention n = 53	Controls N = 63	
Sex	Females	71 (61.2)	32 (60.4)	39 (61.9)	0.866^a^
	Males	45 (38.8)	21 (39.6)	24 (38.1)	
Age, years	Mean (SD)	43.6 (8.7)	42.8 (7.0)	44.3 (9.9)	0.347^b^
ART duration, years	Mean (SD)	4 (1.8)	3.78 (1.8)	4.1 (1.8)	0.289 ^b^
Height, centimetre	Mean (SD)	1.6 (0.1)	1.6 (0.09)	1.6 (1.1)	0.373 ^b^
Weight	Mean (SD)	76.8 (11.9)	79 (11.4)	75 (12.1)	0.071 ^b^
Body mass index, kg/m^2^	Mean (SD)	29.4 (4.4)	29.7 (4.8)	29.0 (4.1)	0.397 ^b^
	25–29.9 kg/m^2^	79 (68.1)	35 (66.0)	44 (69.8)	0.662^a^
	≥30 kg/m^2^	37 (31.9)	18 (34.0)	19 (30.2)	
WC, centimetre	Mean (SD)	97 (9.2)	97.7 (9.6)	96.4 (8.9)	0.462 ^b^
Hip circumference	Mean (SD)	103.9 (8.8)	105.1 (10)	102.9 (7.5)	0.175 ^b^
Waist-hip ratio	Mean (SD)	0.94 (0.097)	0.93 (0.098)	0.94 (0.096)	0.715 ^b^
HDL-cholesterol	Mean (SD)	36.4 (8.3)	35.9 (7.5)	36.7 (9)	0.61^b^
Percent body fat	Mean (SD)	39.7 (4.9)	40.6 (9.6)	39.1 (7.8)	0.364 ^b^
Fat mass index, kg/m^2^	Mean (SD)	11.8 (3.9)	12.39 (4.7)	11.29 (3.0)	0.130 ^b^
VF level (1–30 range)	Mean (SD)	9.97 (2.96)	9.9 (2.5)	10 (3.3)	0.821 ^b^
Fat free mass, kg	Mean (SD)	46.3 (10)	46.7 (8.8)	45.98 (11)	0.698 ^b^
Fat free mass index, kg/m^2^	Mean (SD)	17.3 (2.0)	17.36 (1.9)	17.32 (2.1)	0.928 ^b^
Percent skeletal muscle	Mean (SD)	26.2 (4.9)	26 (5.3)	26.3 (4.6)	0.791 ^b^
Total energy, Kilocalorie	Mean (SD)	2034 (435.3)	2008.7 (398.6)	2056 (466)	0.563 ^b^
Carbohydrate, Kilocalorie	Mean (SD)	1417.3 (306.4)	1371.5 (259.2)	1456 (338.4)	0.14 ^b^
Protein, Kilocalorie	Median (IQR)	211.5 (177–299)	194.6 (169–324)	211 (185–274)	0.673^c^
Fat, Kilocalorie	Median (IQR)	336.4 (238–465)	342 (249.5–485)	323 (230–451)	0.576^c^
Fibres, grams	Mean (SD)	32.8 (13.5)	29.4 (12.7)	35.7 (13.7)	0.012 ^b^

Values are presented as numbers (%) unless otherwise indicated; ART, antiretroviral therapy; HDL, high density lipoprotein; WC, waist circumference; VF, visceral fat; IQR: interquartile range; kg, kilogram; m^2^, square meter; SD, standard deviation; (^a^, significance by chi square test; ^b^, significance by independent *t*-test; ^C^, significance by Mann-Whitney U tests).

### 3.2 Baseline dietary diversity score and food frequency patterns of the study participants

Average DDS among the study participants was 4.1 (±0.97). A substantial proportion, 72.4 %, displayed low dietary diversity, with 4.3 % consuming foods from only two groups, 19.8 % from three groups and 48.3 % from four groups. In contrast, 27.6 % of participants included foods from five or more food groups in their diet. An adequate DDS is characterized by the intake of ≥5 diverse food groups. It was achieved by 26.4 % of participants in the intervention group and 28.6 % in the control group.

In terms of food frequency, over 98 % of participants reported consuming foods made from cereals, grains, and white roots or tubers more than once a day. In comparison, only 3.4 % of participants ate fruits daily, while 14.7 % consumed vegetables on a daily basis. Fish was the least frequently consumed food, with only 0.9 % of participants eating it once per week (Table 2).

**Table 2:** Food frequency patterns of the study participants (n = 116).

Frequency of food item intake	Grains, roots and Tubers (%)	Pulses (%)	Seeds and nuts (%)	Eggs (%)	Dairy products (%)	Meat and meat products (%)	Any vegetables (%)	Any fruits (%)	Fats and oils (%)	Fishes (%)	Sweets and drinks (%)
Never	0.0	0	48.3	52.6	49.1	48.3	8.6	19	0.0	99.1	69.8
Once/week	0.0	0.9	25.9	19.8	8.6	12.9	14.7	16.4	0.0	0.9	0.9
Two times/week	0.0	2.6	13.8	16.4	13.8	23.3	25.9	35.3	0.0	0.0	11.2
3-4 times/week	0.0	1.7	8.6	6.0	10.3	10.3	35.3	21.6	0.0	0.0	4.3
5-6 times/week	0.0	0.9	1.7	0.0	0.9	1.7	8.6	4.3	0.0	0.0	1.7
Once/day	1.7	6.9	0.9	5.2	12.9	3.4	14.7	3.4	0.0	0.0	10.3
>1 times/day	98.3	87.1	0.9	0.0	4.3	0.0	0.9	0.0	99.1	0	1.7
Total (%)	100	100	100	100	100	100	100	100	100	100	100

### 3.3 Changes after six-month intervention

Table 3 presents the intragroup comparisons of variables after six months. Intervention group showed average 0.6 kg (95 %CI -1.3, 0.11; p = 0.11) weight loss from their baseline, whereas control participants gained 0.8 kg (95 %CI 0.27, 1.3; p = 0.004) weight from their baseline. WC and HC in the intervention group showed significant reductions from their baseline (p < 0.05). Significant change in BFP, FM and FMI was observed in the counselled group: BFP decreased by 1.4 % (95 %CI -2.0, −0.86, p < 0.001), FM by 1.38 kg (95 %CI -2.1, −0.6, p = 0.001) and FMI by 0.54 kg/m^2^ (95 %CI -0.85, −0.24, p ​= ​0.001) from baseline. In contrast, the controls showed significant increases in BFP (+0.6 ​% [95 ​% CI 0.12, 1.1, p ​= ​0.01]), FM (+0.78 kg [95 %CI 0.27, 1.3, p ​= ​0.003]), FMI (+0.3 ​kg/m^2^ [95 %CI 0.1, 0.5, p ​= ​0.004]) and visceral fat (+0.28 [95 %CI 0.09, 0.48, p ​= ​0.005]) from their baseline.

**Table 3:** Changes in body composition and blood pressure from baseline to six-month intervention.

Outcome variables	Changes within group^a^					
	Intervention group (n = 53)			Control group (n = 63)		
	At 6 month Mean (SD)	Δ from baseline Mean (95 %CI)	p-value	At 6 month Mean (SD)	Δ from baseline Mean (95 %CI)	p-value
Weight, kg	78.5 (11.7)	−0.6 (-1.3, 0.11)	0.10	75.98 (11.97)	+0.8 (0.27, 1.3)	0.004
Body mass index, kg/m^2^	29.53 (4.8)	−0.23 (-0.49, 0.03)	0.078	29 (2.9)	−0.048 (-0.09, 0.8)	0.94
Waist circumference, cm	96.35 (9.6)	−1.3 (-2.2, −0.45)	0.004	95.87 (8.9)	−0.53 (-1.2, 0.15)	0.13
Hip circumference, cm	103.46 (9.9)	−1.7 (-2.4, −0.9)	<0.0001	102.11 (7.4)	−0.79 (-1.5, −0.1)	0.02
%Body fat	39.02 (9.3)	−1.4 (-2.0, −0.86)	<0.0001	39.66 (7.6)	+0.6 (0.12, 1.1)	0.01
Fat mass, kg	30.95 (10)	−1.38 (-2.1, −0.6)	0.001	29.83 (6.2)	+0.78 (0.27, 1.3)	0.003
Fat mass index, kg/m^2^	11.8 (4.6)	−0.54 (-0.85, −0.24)	0.001	11.59 (2.97)	+0.3 (0.1, 0.5)	0.004
Visceral fat (1–30 range)	9.66 (2.5)	−0.24 (-0.59, 0.1)	0.15	10.32 (3.3)	+0.28 (0.09, 0.48)	0.005
Fat free mass, kg	47.5 (8.5)	+0.84 (0.18, 1.5)	0.014	46.15 (10.9)	+0.17 (-0.2, 0.5)	0.37
Fat free mass index, kg/m^2^	17.69 (1.9)	+0.34 (0.08, 0.6)	0.01	17.39 (2.0)	+0.06 (-0.08, 0.2)	0.38
% Skeletal muscle	26.77 (5.1)	+0.9 (0.51, 1.31)	<0.0001	25.92 (4.4)	−0.34 (-0.8, 0.06)	0.096
Systolic BP(mmHg)	125 (17.7)	−3.1 (-6.3, 0.2)	0.065	124.3 (21.7)	−1.4 (-4.9, 2.2)	0.44
Diastolic BP(mmHg)	81.2 (10.9)	−3.7 (-6.2, −1.2)	0.004	80.2 (13.1)	−1.4 (-4.6, 1.8)	0.39

BP,blood pressure; CI, confidence interval; cm, centimetre; mmHg, millimetre of mercury; SD, standard deviation.

a Changes in variables within group was assessed by paired *t*-test.

The intergroup comparison of differences over six months revealed significant changes among participants in the intervention group compared to the controls. Participants in the counselling group lost substantial weight (−1.4 kg [95 %CI -2.2, −0.53]), BFP (−2.02 % [95 %CI -2.7, −1.3]), FM (−2.16 kg [95 %CI -3.1, −1.3]), FMI (−0.847 kg/m^2^ [95 ​% CI -1.2, −0.495]), and VF (−0.53 [95 ​% CI -0.904, −0.157]) compared to controls at 6 months. In addition, the counselled group showed greater improvement in percentage skeletal muscle mass than controls (+1.27 ​% [95 %CI: 0.68, 1.8]). Cohen's d values for differences were indicated as follows: for weight, d ​= ​−0.6 (medium effect); for BFP, d ​= ​−1.03 (large effect); for FM, d ​= ​−0.889 (large effect); for VF, d ​= ​−0.525 (medium effect); and for percentage skeletal muscle mass, d ​= ​0.8 (large effect) (Table 4).

**Table 4:** Effect size of intervention on anthropometrics, body composition and blood pressure.

Between group difference (Intervention group Vs. Controls).^a^			
Outcome variables	Δ from Control	Significance	Effect size
	Mean (95 %CI)	p-value	Cohen's d (95 % CI)
Weight, kg	−1.4 (-2.2, −0.53)	0.002	−0.6 (-0.973, −0.266)
Body mass index, kg/m^2^	−0.18 (-1.7, 0.80)	0.71	−0.068 (-0.43, 0.297)
Waist circumference, cm	−0.8 (-1.9, 0.29)	0.148	0.271 (-0.638, 0.096)
Hip circumference, cm	−0.876 (-1.9, 0.13)	0.087	−0.322 (-0.689, 0.047)
Body fat (%)	−2.02 (-2.7, −1.3)	<0.0001	−1.03 (-1.42, −0.642)
Fat mass, kg	−2.16 (-3.1, −1.3)	<0.0001	−0.887 (-0.127, −0.503)
Fat mass index, kg/m^2^	−0.847 (-1.2, −0.495)	<0.0001	−0.887 (-1.27, −0.502)
Visceral fat (1–30 range)	−0.53 (-0.904, −0.157)	0.006	−0.525 (-0.895, −0.152)
Fat free mass, kg	+0.673 (-0.05, 1.39)	0.068	0.34 (-0.025, 0.711)
Fat free mass index, kg/m^2^	+0.27 (-0.007, 0.55)	0.056	0.36 (-0.009, 0.728)
Skeletal muscle (%)	+1.27 (0.68, 1.8)	<0.0001	0.80 (0.42, 1.18)
Systolic BP(mmHg)	−1.7 (-6.5, 3.1)	0.49	−0.129 (-0.494, 0.237)
Diastolic BP(mmHg)	−2.3 (-6.5, 1.8)	0.267	−0.21 (-0.574, 0.159)

BP,blood pressure; CI, confidence interval; cm, centimetre; kg, kilogram; mmHg, millimetre of mercury.

a Change score in variables between groups assessed by independent *t*-test; Effects size based on Cohen's d: <0.19 for insignificant; 0.20–0.49 for small; 0.50–0.79 moderate and >0.80 for large.

### 3.4 Correlations between counselling and changes in weight and body composition

Counselling was significantly correlated with reduction in weight, resulting in a mean weight loss of 1.43 kg (β = −1.43, 95 % CI: 2.4, −0.45). Counselling also was correlated with an average decrease of 1.76 % in BFP (β = − 1.76, 95 % CI: 2.5, − 0.97) and an increase of 1.28 % in percentage skeletal muscle mass (β = 1.28, 95 % CI: 0.67, 1.9), suggesting a a favourable effect on muscle composition (Table 5).

**Table 5:** Linear regression model for associations of intervention with changes in weight and body composition.

Baseline variables	Body weight (Δ)		%body fat (Δ)		%skeletal muscle (Δ)	
	β(95 % CI)	P-value	β(95 % CI)	P-value	β(95 % CI)	P-value
Constant	2.4 (-4.3, 9.1)	0.481	5.8 (0.39, 11.2)	0.036	−0.26 (-3.9, 3.4)	0.887
Intervention	−1.43 (-2.4, −0.45)	0.005	−1.76 (-2.5, −0.97)	<0.0001	1.28 (0.67, 1.9)	<0.0001
Baseline weight	−0.006 (-0.05,0.03)	0.767	−0.02 (-0.05, 0.016)	0.703	−0.003 (-0.03, 0.02)	0.80
Age	−0.01 (-0.07, 0.05)	0.725	−0.028 (-0.08, 0.02)	0.318	0.03 (-0.08, 0.07)	0.123
Alcohol intake	0.15 (-0.12, 0.93)	0.779	−0.06 (-0.93, 0.81)	0.757	−0.05 (-0.72, 0.63)	0.893
ART duration	0.07 (-0.19, 0.34)	0.578	0.03 (-0.19, 0.24)	0.80	−0.07 (-0.24, 0.09)	0.393
DTG-based regimen	1.1 (-1.2, 3.4)	0.338	−0.54 (-2.4, 1.36)	0.569	0.45 (-0.99, 1.9)	0.538
Baseline Carbs calorie	−0.001 (-0.003, 0.00)	0.148	0.0 (-0.002, 0.001)	0.867	0.001 (-0.001, 0.002)	0.322
Baseline Protein calorie	0.00 (-0.003, 0.004)	0.780	−0.001 (-0.004, 0.002)	0.457	∗	–
Baseline Fat calorie	0.003 (0.0, 0.007)	0.086	0.2 (-0.69, 1.1)	0.659	0.001 (-0.001, 0.004)	0.222
Baseline fibers intake	0.018 (-0.03, 0.06)	0.418	0.01 (-0.02, 0.05)	0.539	−0.014 (-0.04, 0.01)	0.298
Baseline dietary diversity	−0.5 (-1.6, 0.63)	0.393	0.18 (-0.70, 1.06)	0.686	0.24 (-0.44, 0.92)	0.484
Baseline %body fat	−0.02 (-0.08, 0.04)	0.437	−0.05 (-0.1, −0.005)	0.031	∗	–
Baseline % skeletal muscle	∗	–	∗	–	−0.08 (-0.24, −0.02)	0.013
Baseline systolic BP	0.006 (-0.03, 0.04)	0.731	0.03 (0.007, 0.06)	0.016	−0.02 (-0.04, −0.001)	0.044
Baseline diastolic BP	−0.017 (-0.07, 0.03)	0.513	−0.06 (-0.1, −0.017)	0.006	0.04 (0.008, 0.07)	0.014
Baseline triglyceride level	0.001 (-0.005, 0.007)	0.696	0.003 (-0.002, 0.008)	0.2183	−0.004 (-0.008, −0.001)	0.027

Δ, change from baseline; DTG, dolutegravir; variance inflation factor for body weight ​= ​8.7; % body fat ​= ​8.7 ​% skeletal mass ​= ​8.6; ∗ excluded variable with a variance inflation factor of ≥10.

After adjusting for covariates, significant interaction effects of “group × time“ was found for variables such as weight (β = −1.1, 95 %CI -1.9, −0.3, p = 0.006), BFP (β = −1.8, 95 %CI -2.5, −1.1, p < 0.0001), FFM (β = 0.7, 95 %CI 0.18, 1.2, p = 0.009), and percentage skeletal muscle mass (β = 1.2, 95 %CI 0.63, 1.8, p < 0.0001).

Overall, 33.6 % of participants showed a reduction of ≥1 % of their baseline weight, with 43.4 % in the intervention group and 25.4 % in the control group (p = 0.041). In addition, 18.1 % lost at least 3 % of their body weight from baseline. Of these, 24.5 % participants were from the intervention group and 12.7 % were from controls. Multiple linear regression analysis showed that counselling had a significant effect on achieving at least a 3 % reduction in weight from baseline (Table 6).

**Table 6:** Association of interventions with ≥3 % weight loss among the study participants.

Variables	95 % Confidence interval for β			
	β	Lower limit	Upper limit	p-value
(Constant)	00.711	−00.420	1.842	0.215
Intervention	00.158	00.002	00.314	0.048
Females	−00.028	−00.204	00.148	0.754
Age >40 years	00.041	−00.207	00.288	0.746
Receiving dolutegravir based regimen	−00.055	−00.435	00.324	0.773
High density lipoprotein cholesterol	00.005	−00.005	00.014	0.309
Baseline protein intake (calorie)	00.000	−00.001	00.000	0.181
Baseline adequate diversified dietary intake	00.033	−00.148	00.213	0.721
Baseline fat intake (calorie)	00.000	−00.001	00.000	0.426
Baseline fibre intake (gram)	00.001	−00.005	00.007	0.710
Waist circumference (centimeter)	−00.006	−00.017	00.005	0.266
Body mass index (kilogram/metre^2^)	−00.001	−00.022	00.021	0.949

β, unstandardized coefficient.

## 4 Discussion

This study assessed the impacts of counselling on weight and body composition variables. The counselling included one in-person session at randomization, followed by at least two telephone sessions within two months of intervention. Over six months, participants in the counselling group led to substantial decrease in weight (−1.4 kg), BFP (−2.0 %) and FM (−2.16 kg) compared to the controls. Additionally, considerable increase in percentage skeletal muscle (+1.27 ​%) and FFM (+0.67 kg) were observed among participants in the counselling group when compared to controls. Lifestyle modification counselling was also showed significantly correlation with achieving a minimum weight loss of 3 %.

Participants who received counselling experienced a significant decrease in weight (−1.4 kg) over six months compared to the control group. The finding is consistent with previous studies showing similar results with face-to-face, remote or combined counselling interventions [[44], [45], [46], [47]]. Additionally, meta-analysis studies have demonstrated that increased counselling intensity significantly improves weight loss outcomes [48,49].

Studies have shown that lifestyle interventions combining dietary modifications and regular physical activity are effective for weight loss and obesity management [18,50]. However, the intragroup comparison in our counselling group indicated small weight loss from baseline, contrary to reports of other studies [51,52]. This may be attributed to the considerable increase in FFM and percentage skeletal muscle in our study, which may have replaced weight loss and influenced the weight loss results.

We found significant reductions in BFP (−1.4 %) and FM (−1.38 kg) in the counselled group from their baseline. Additionally, the intervention group showed significant reductions in BFP (−2.0 %), VF (−0.53), and FM (−2.16 kg) compared to the control group. These results are consistent with findings from several studies conducted among individuals with chronic health conditions, the general population [[53], [54], [55]], and PLWH [56,57].

PLWH on ART may be at increased risk of subcutaneous and visceral fat accumulation, contributing to central obesity and potentially increasing the risk of certain cancers through metabolic and inflammatory pathways [58], as well as elevating metabolic and cardiovascular risks [59]. Therefore, non-pharmacological treatments such as lifestyle interventions are essential for promoting healthy weight loss and reducing the impact of obesity among individuals with obesity [17,18]. Additionally, we observed a significant increase in FM (+0.78 kg) and VF levels (+0.28) in the control group from their baseline. This finding aligns with previous studies that reported similar results [60]. Inadequate physical exercise in PLWH compared to those without HIV, may contribute to alterations in body composition including the accumulation of central fat [61].

Studies have shown improvements in skeletal muscle mass, with a significant increase in the intervention group compared to the controls [[62], [63], [64]]. Consistent with these findings, we observed a substantial increase in percentage skeletal muscle in the counselled group from their baseline (+0.91) and compared to the control group (+1.27). Dietary modifications [65], along with increased exercise among participants in the counselling group may have contributed to this improvement in skeletal muscle [63,65].

In this study, participants in the counselling group showed a significant increase in FFM over six months from baseline; however, this increase was not significantly different from that in the control group. These findings are consistent with previous studies reporting increases in FFM among individuals with overweight or obesity who received lifestyle interventions [49,66]. Improvements in physical activity and dietary habits following counselling may promote the progress of skeletal muscle and increases in 10.13039/100018375FFM [67].

In linear regression, the intervention was significantly correlated with changes in weight, BFP and percentage skeletal muscle mass after adjusting for baseline factors. Additionally, intervention was significantly correlated with ≥3 % weight reduction from baseline. Similar results have been reported in several studies, which demonstrating the substantial influence of interventions on body weight reduction [68,69]. This may have clinically significant implications for improving cardiometabolic risk markers [17,18,22]. Consequently, offering routine and successful lifestyle counselling could ensure that at-risk individuals receive timely and may help reduce obesity related health problems [70].

## 5 Limitations

This study had several limitations. This study focused solely on adults with overweight and obesity, which may have restricted the applicability of the findings to the general population. We did not assess cardiovascular disease indicators, such as lipid profiles, or metabolic indicators like fasting blood sugar at the completion follow up, which may limit our evaluation of metabolic and cardiovascular outcomes. Nearly one-fourth of participants in the intervention group reported economic constraints, which may have limited their access to a variety of healthy, diversified, and fiber-enriched foods essential for supporting weight loss and management. Additionally, the baseline data on food frequency, 24-h ​dietary recall, and physical exercise were self-reported, which could have introduced inaccuracies and response bias.

## 6 Conclusion

This study demonstrates that lifestyle counselling led to significant changes in weight and body composition over six months in adults with overweight or obesity receiving ART. Additionally, lifestyle modification interventions were notably associated with changes in weight, BFP, percentage skeletal muscle mass and a weight loss of ≥3 % from baseline. Therefore, the routine provision of lifestyle counselling for individuals with overweight or obesity on ART could help mitigate obesity-related health risks and enhance clinical outcomes. In addition, well-controlled trials with larger sample size and sex stratifications are essential to assess the long-term effects of lifestyle interventions on cardiometabolic risk factors in this population.

## Clinical key take away messages

• Lifestylecounselling resulted in significant reductions in weight, FM, VF, and BFP.
• The counselled group showed an increase in FFM and skeletal muscle mass.
• Lifestyle counselling correlated with achieving ≥3 % weight loss.

## Declaration of artificial intelligence (AI) and AI-assisted technologies

The authors did not use AI tools during the preparation of this work.

## Author contributions

Agete Tadewos Hirigo, Zelalem Debebe, Daniel Yilma and Ayalew Astatkie conceptualized and designed the study. Agete Tadewos Hirigo and Moges Derbe contributed to data collection. Agete Tadewos Hirigo, Zelalem Debebe, Daniel Yilma and Ayalew Astatkie conducted data analysis and drafted the initial manuscript. Zelalem Debebe, Daniel Yilma and Ayalew Astatkie provided supervision, interpreted the data, critically revised the manuscript and gave final approval for publication. All authors reviewed and approved the final manuscript

## Ethics review

The study protocol was approved by the Institutional Review Board of the College of Natural and Computational Sciences at Addis Ababa University (Approval No: IRB/07/14/2022). All participants were provided with information regarding the research objectives and potential benefits, and written informed consent was obtained.

## Trial registration

This clinical trial was retrospectively registered with the Thai Clinical Trials Registry on September 5, 2024 (TCTR20240905007) and can be accessed at https://www.thaiclinicaltrials.org/show/TCTR20240905007.

## Declaration of competing interest

The authors declare that they have no known competing financial interests or personal relationships that could have appeared to influence the work reported in this paper.
